# Moyamoya Disease Associated With Morning Glory Disc Anomaly and Other Ophthalmic Findings: A Mini-Review

**DOI:** 10.3389/fneur.2020.00338

**Published:** 2020-05-15

**Authors:** Yue-ye Wang, Ke-yao Zhou, Yang Ye, Fan Song, Jin Yu, Jin-cao Chen, Ke Yao

**Affiliations:** ^1^Eye Center, Second Affiliated Hospital of Zhejiang University School of Medicine, Hangzhou, China; ^2^Department of Neurosurgery, Zhongnan Hospital of Wuhan University, Wuhan, China

**Keywords:** moyamoya disease, morning glory disc anomaly, neuro-ophthalmology, ocular findings, review

## Abstract

Moyamoya disease (MMD) is a chronic cerebrovascular disease that frequently results in intracranial ischemia or hemorrhage. Its concurrence with varying ophthalmic findings is relatively rare yet may lead to irreversible blindness. We performed a search and review of the literature to characterize the relevance of MMD (excluding moyamoya syndrome) and ophthalmic findings. As a result, a total of 38 articles identified from PubMed and Web of Science were included in this mini-review. Patients with MMD sometimes present with decreased visual acuity or visual field defects before the onset of symptomatic cerebrovascular dysfunction. The most predominant ophthalmic condition in MMD patients is the morning glory disc anomaly (MGDA). Deficiency during neuroectodermal genesis and subsequent mesodermal changes may be responsible for the association between these two diseases. Thus, it may be beneficial for patients with MGDA to receive cerebral vascular examinations as the precaution against life-threatening intracranial angiopathy. Other ophthalmic findings reported in cases of MMD include retinal vascular occlusion, optic disc pallor, cortical blindness, etc. For most of the patients with MMD, retinal examinations would be recommended to prevent potential loss of vision. It is essential for both neurologists and ophthalmologists to be aware of the correlation between cerebrovascular diseases such as MMD and ocular manifestations to achieve a comprehensive diagnosis.

## Introduction

Moyamoya disease (MMD) is a life-threatening cerebrovascular disease that sometimes may also lead to irreversible vision loss. The disease is characterized as progressive steno-occlusive changes occurring at the terminal portion of the internal carotid artery (ICA), initial middle cerebral artery (MCA), and anterior cerebral artery (ACA) (1). Its typical appearance on angiography of an abnormal collateral arterial network at the base of the skull resembles a puff of smoke on angiography, which is described as “moyamoya” in Japanese. MMD is an idiopathic disease, and otherwise, when patients are with moyamoya angiopathy, which is in association with other underlying diseases, e.g., neurofibromatosis or hyperthyroidism, they would be categorized as having moyamoya syndrome (2).

Previous epidemiological studies show that the areas with high morbidity incidence of MMD are mainly East Asian countries, such as Japan, China, and Korea. According to one study in Taiwan, the prevalence of MMD in 2011 was 1.61 (per 100,000) (3). There are two peaks of the incidence, respectively, at the age of 10–20 and 35–50 (4). Ischemia and hemorrhage are predominant consequences of MMD, which mostly occur in the territories of the ICA and MCA, and sometimes present as visual symptoms onset (2). MMD relies on cerebral angiographic examinations for diagnosis and surgical revascularization for treatment, the most widely applied extracranial–intracranial bypass, for instance.

Independent ophthalmic presentations are uncommon in MMD. However, several cases of morning glory disc anomaly (MGDA) have been reported in pediatric MMD patients. The typical intracranial vascular and retinovascular anomalies suggest that there may be a common denominator between these two diseases (5). Central retinal artery occlusion, amaurosis fugax, and ocular ischemic syndrome were also reported in patients with MMD (6).

Patients who complain of visual dysfunction may be unlikely to get initially diagnosed as having potential cerebral vascular disease. Since ocular findings are usually neglected in MMD, none of those cases have been reviewed systematically. Herein, we conduct a mini-review on MMD-associated ophthalmic manifestations to provide an overall insight into its possible clinical relevance and investigate hypotheses regarding its mechanism. Since moyamoya syndrome is defined as being accompanied by correlative conditions and it would be controversial to distinguish the direct effect of this intracranial angiopathy on ophthalmic changes, we have not included any of the literature on moyamoya syndrome in this study.

## Literature Search

We performed a search of the literature in the PubMed and Web of Science databases to identify articles related to MMD and ophthalmic findings on September 15th, 2019. The titles and abstracts of those articles were reviewed by two reviewers to confirm their quality and eligibility for further examination. The inclusion criteria were as follows: (1) MMD and ocular manifestations were simultaneously mentioned in the title or abstract, and (2) original studies or case reports. The exclusion criteria were as follows: (1) without a definite diagnosis of MMD; (2) non-English article; (3) Moyamoya syndrome; and (4) with a non-ophthalmic disease, which could probably affect visual function and not conforming to the diagnostic criteria of moyamoya syndrome. MMD was diagnosed when there was no specific underlying disease, including genetic, hereditary disorders, hematological disorders, connective-tissue diseases, infectious or chronic inflammatory conditions, metabolic diseases, and vascular injury in this study. Based on the inclusion and exclusion criteria, a total of 38 eligible articles were identified and reviewed, and the year of publication ranged from 1981 to 2019.

All articles were classified according to the ophthalmic diagnosis. The various ophthalmic findings associated with MMD in these articles included MGDA, retinal vascular occlusion, nystagmus, amaurosis fugax, hypertropia, diplopia, decreased vision, visual field defect, iris hypoplasia, retinal artery tortuosity, ocular ischemia, retinochoroidal atrophy or coloboma, anterior ischemic optic neuropathy, optic nerve coloboma, optic nerve pallor, optic glioma, congenital cataract, and congenital glaucoma.

## MMD and Various Ophthalmic Findings

### Morning Glory Disc Anomaly

According to our review, MGDA is the most frequent ophthalmic manifestation in all MMD patients included. MGDA is a rare congenital deficiency of the optic disc characterized by an enlarged, funnel-shaped excavation of the posterior pole involving the hypogenetic optic disc, which resembles a morning glory flower. Generally, this malformation is considered to be unilateral, while bilateral involvements were occasionally reported. Patients of MGDA mainly present with decreased visual acuity at the onset due to dysplasia of the optic disc. This disease is associated with various conditions, including intracranial vascular abnormalities; mid-line facial abnormalities; 47, XYY syndrome; Down syndrome; etc. (7–9).

We identified 13 articles of MMD-associated MGDA, including 11 case reports, 1 ophthalmic image report, and 1 retrospective study (5, 9–20). The retrospective study evaluated intracranial vascular anomalies in 20 patients with MGDA and diagnosed four of them with MMD (9). The ophthalmic images published in *JAMA Ophthalmology* in 2015 showed fundus and ultrasonography of an MGDA patient. The compensatory collateralization of chorioretinal anastomoses in this patient also imparted a “moyamoya” bypass system, indicating that MGDA may have pathological correlations with MMD (11).

The characteristics of the 11 cases are listed in (Table 1). Of all the patients included, 6 (54.5%) were male; most of them were children, with a mean age of 9.7 years (median 5, range 2–29). A total of 9 (81.8%) patients were diagnosed with unilateral MGDA, while only 2 (18.2%) were diagnosed with bilateral MGDA. Among all the 8 (72.7%) bilateral MMD patients, 7 had unilateral MGDA (left eye: 5, right eye: 2), and only one had bilateral MGDA. The remaining two patients with unilateral MMD were associated with ipsilateral MGDA, while the other patient with unilateral MMD was associated with bilateral MGDA. Generally, MDGA is unilaterally presented, while MMD is bilaterally presented, yet the severity can differ between sides. Therefore, it would be logical that the most frequent situation in patients is with bilateral MMD and unilateral MGDA. Interestingly, patients with unilateral MMD only presented MGDA on the same side in current cases, the situation of these two diseases with opposite laterality has not been described yet. Besides, 3 (27.3%) patients were found with midline cranial defects, 2 (18.2%) patients with choroidal coloboma, and 2 (18.2%) patients with meningoencephalocele. The involvement of ICA in cerebral angiography was most prevalent, found in 10 (90.9%) patients, while MCA involvement was found in 6 (54.5%) patients and ACA involvement was found in 3 (27.3%) patients.

**Table 1:** Cases of Moyamoya disease associated with morning glory disc anomaly.

**No**.	**Author**	**Year**	**Age**	**Gender**	**Diagnosis**	**Intracranial vessels**
**Case 1**	Sathyan, S.	2018	16	Female	MGDA (OS); high myopia (OS); bilateral MMD	Left ICA stenosis; (right side post-operation)
**Case 2**	Ponnatapura, J.	2018	4	Male	MGDA (OS); bilateral MMD	Bilateral ICA stenosis; left ACA occlusion
**Case 3**	Loddenkemper, T.	2008	2	Female	MGDA (OU); bilateral MMD; pituitary stalk duplication	Bilateral ICA and MCA stenosis; right ACA stenosis
**Case 4**	Williams, M.	2006	16	Female	MGDA (OS); bilateral MMD	Bilateral ICA stenosis
**Case 5**	Sabti, K.	2005	3	Male	MGDA (OU); left MMD; Congenital third nerve palsy	Left ICA stenosis
**Case 6**	Quah, B. L.	2005	4	Male	MGDA (OS); bilateral MMD; midline cranial defects	Right ICA and MCA stenosis; left ICA occlusion
**Case 7**	Taskintuna, I.	2003	4	Male	MGDA (OD); bilateral MMD; persistent hyaloid artery remnant; midline cranial defects	Bilateral ICA stenosis; bilateral MCA M1 segment stenosis and occlusion
**Case 8**	Krishnan, C.	2000	14	Female	MGDA (OD); right MMD choroidal coloboma	Right ICA and MCA stenosis
**Case 9**	Komiyama, M.	2000	29	Male	MGDA (OS); bilateral MMD; congenital cataract (OD); basal meningoencephalocele; panhypopituitarism	Bilateral ICA occlusion
**Case 10**	Bakri, S. J.	1999	10	Female	MGDA (OD); bilateral MMD; chorioretinal coloboma (OS); sphenopharyngeal meningoencephalocele	Bilateral MCA stenosis
**Case 11**	Massaro, M.	1998	5	Male	MGDA (OS); left MMD; retinal detachment (OS)	Left ICA, and its bifurcation into MCA and ACA stenosis

*MMD, Moyamoya disease; MGDA, morning glory disc anomaly; OS, oculus sinister; OD, oculus dexter; OU, oculus uterque; ICA, internal carotid artery; MCA, middle cerebral artery; ACA, anterior cerebral artery*.

Most of the patients in (Table 1) were also diagnosed with other congenital abnormalities such as midline cranial defect or duplication of the pituitary stalk, reflecting mutual fetal dysgenesis to be the possible underlying cause. Komiyama, M once postulated that MMD is a vascular form of neurocristopathy. The finding that the steno-occlusive changes of MMD only exist in the cerebral arteries originating from the neural crest fits in this context (21). According to the mainstream hypothesis, MGDA is the consequence of primary neuroectodermal dysgenesis, characteristically with central gliosis and vascular damage at the optic disc (22). The pathological changes involving the optic disc and intracranial vessels are suggestive of a similar deficiency during ectoderm development in both diseases.

The risk of intracranial vascular anomalies was higher in MGDA patients than in non-MGDA patients, according to Lenhart's study (9). This study reviewed and evaluated neurologic anomalies in 20 MGDA patients and 40 non-MGDA patients as a control. As a result, cerebral anomalies were more common in patients with MGDA, 9 of 20 (45%) compared with 10 in 40 (25%) in the control group. The intracranial vascular anomalies in these patients ranged within a spectrum, from only mild congenital variants to progressive stenosis or occlusion, as the most severe condition to be MMD. MMD was diagnosed in 3 of 20 patients with MGDA, while it was diagnosed in none of the control. The estimated prevalence of MMD in MGDA patients was 15% in this study, significantly higher than that in the whole population. Nevertheless, the prevalence in this study is deduced within a limited sample size, and further study on a larger scale is still required.

Interestingly, a cross-sectional study analyzed the retinal morphology of MMD patients by optical coherence tomography. In patients without symptomatic retinal abnormalities, a significant reduction in optic nerve head volume and a thinner macular retinal nerve fiber layer were revealed, similar to the characteristics of MGDA. The authors indicated that both MMD and MGDA might involve neuroectodermal dysgenesis and subsequent mesodermal changes (23), even if it was not able to be distinguished in fundus photography or diagnosed as retinopathy, the volume of optic nerve head was with detectable change. Abnormal retinal findings in MGDA may be indications of intracranial vascular pathogenesis as the consequence of mutual mesenchymal dysgenesis, and optical coherence tomography is a simple and non-invasive technique to achieve early diagnosis precede the clinical manifestation of MMD. Patients with decreased optic nerve head volume or thinning macular retinal nerve fiber layer should receive neuroradiological examinations for the diagnosis of any potential cerebral vascular disease. Those patients have not been conclusively shown to have the same risk for intracranial vascular occlusion, though the association should not be underestimated. Most of them may only present with mild vascular anomalies. Thus, the indication of preemptive treatment with intracranial vascular bypass is limited in this subgroup, and further discussion with a neurosurgeon would be recommended.

### Retinal Vascular Occlusion

Obstruction of retinal vessels, particularly the central retinal artery, has been reported in several cases of MMD patients. A total of 6 cases reporting MMD associated retinal vascular occlusion are reviewed in this study (Table 2) (24–29). The chief complaint of almost all patients was acute vision loss. One patient also complained of a severe headache, while two patients were found with a relative afferent pupillary defect. The age of those patients ranged from 25 to 40 years old, in accordance with the second age peak of onset of MMD.

**Table 2:** Cases of Moyamoya disease associated with retinal vascular occlusion and other ophthalmic findings.

**No**.	**Author**	**Year**	**Age**	**Gender**	**Diagnosis**	**Intracranial vessels**
**Retinal vascular occlusion**						
**Case 12**	Guclu, H.	2016	36	Male	Consecutive BRVO (OU); bilateral MMD;	Bilateral ICA occlusion
**Case 13**	Garoon, R.	2015	41	Female	CRVO (OS); macular edema; bilateral MMD	Bilateral ICA stenosis
**Case 14**	Kumar, M. A.	2013	26	Male	CRAO (OD); right MMD	Right ICA occlusion
**Case 16**	Ushimura, S.	1993	31	Female	CRAO (OD); bilateral MMD; mitral valve prolapse	Bilateral ICA occlusion
**Case 17**	Chace, R.	1984	NA	NA	Acute retinal artery occlusion	NA
**Case 18**	Slamovits, T. L.	1981	45	Female	CRVO (OS); macular edema; bilateral MMD	Bilateral ICA occlusion
**Ischemia and hemorrhage related visual dysfunction**						
**Case 19**	Nakayama, C.	2019	23	Female	Epileptic nystagmus; right MMD; cerebral blood flow decrease	Right ICA stenosis
**Case 20**	Donggyu Cho	2019	48	Female	Trochlear nerve palsy; bilateral MMD; cerebral hemorrhage	Right MCA occlusion
**Case 21**	Sajja, A.	2017	16	Female	Cortical vision loss; bilateral MMD; bilateral occipital ischemia	Bilateral ICA occlusion
**Case 22**	John, D.	2016	14	Male	TIA; optic disc pallor (OU); arteriolar attenuation (OU); bilateral MMD	Bilateral ICA, ACA, MCA stenosis; basilar and bilateral PCA involvement
**Case 23**	Borah, P.	2014	20	Female	Amaurosis fugax (OD); bilateral MMD	Bilateral ICA, ACA, MCA stenosis
**Case 24**	Kim, Dal-Soo	2007	33	Male	Bilateral MMD; transient blindness (OU); cerebral infarction (right frontal lobe and left occipital lobe)	Bilateral ICA occlusion; bilateral PCA steno-occlusive changes
**Case 25**	Chu, M. K.	2001	30	Female	Transient visual field defect (OU); bilateral MMD; perfusion defect in bilateral temporal, occipital lobes, parietal area	Bilateral ICA occlusion; right PCA stenosis
**Case 26**	Noda	1987	28	Male	Vision decrease; bilateral MMD; central scotoma (OU); cerebral infarction	Abnormal basilar network; bilateral carotid artery occlusion
			62	Male	Homonymous hemianopsia; transient diplopia (OU); bilateral MMD; cerebral infarction	Abnormal basilar network; bilateral carotid artery occlusion
			34	Male	Homonymous hemianopsia; optic disc pallor (OU) bilateral MMD; cerebral infarction	Abnormal basilar network; bilateral carotid artery occlusion
**Other ocular findings**						
**Case 27**	Katsman, D.	2016	30	Female	Retinal Arterial Tortuosity; bilateral MMD	Bilateral MCA stenosis; vascular tortuosity (left anterior inferior cerebellar and left posterior cerebral arteries)
**Case 28**	Papavasileiou, E.	2015	51	Female	Retinal vasculitis (OU); macular edema (OU); bilateral MMD	Right ICA occlusion; left ACA occlusion
**Case 22**	John, D.	2016	13	Male	Optic disc pallor (OU); arteriolar attenuation (OU); bilateral MMD; bilateral occipital lobe ischemia	Supraclinoid ICA and PCA stenosis
			2	Male	Mature cataract (OU); optic disc pallor (OU); bilateral MMD	Bilateral ICA stenosis; bilateral PCA and terminal basilar occlusion
**Case 29**	Verdure, P.	2012	13	Female	Retinochoroidal coloboma (OU); bilateral MMD; acute ischemic stroke in ACA territories	Bilateral ICA hypoplasia; distal ICA stenosis; left MCA stenosis; left PCA occlusion
**Case 30**	Das, D.	2010	25	Female	Optic nerve involvement; transient vision loss; bilateral MMD	Bilateral MCA stenosis; right ICA, PCA stenosis
**Case 31**	Johnson, S. M.	2009	16	Female	Congenital glaucoma; bilateral MMD; myopia	Bilateral MCA; left PCA involvements
**Case 32**	Spengos, K.	2008	42	Female	Optic Nerve Coloboma (OS); left MMD	Anterior and posterior cerebral circulation
**Case 33**	Chen, C. S.	2007	51	Female	Non-arteritic anterior ischemic optic neuropathy; bilateral MMD	Right ICA occlusion; left ICA stenosis
**Case 34**	Khan, N.	2004	4	Female	Iris hypoplasia and fixed dilated pupils (OU); bilateral MMD; bilateral dolichoectatic internal carotid arteries; patent ductus arteriosus	Bilateral ICA stenosis; left ACA occlusion
			6	Male	Iris hypoplasia and fixed dilated pupils (OU); bilateral MMD; patent ductus arteriosus	Bilateral ICA stenosis; left MCA stenosis; left ACA aplasia
**Case 35**	Harissi-Dagher, M.	2004	20	Female	Chorioretinal atrophy (OU); bilateral MMD	Bilateral ICA occlusion
**Case 26**	Noda	1987	6	Male	Amaurosis fugax (OU); concentric construction of visual field (OU) bilateral MMD	Abnormal basilar network; bilateral carotid artery occlusion
**Case 36**	Okuno, T.	1985	5	Female	Optic glioma (OU); bilateral MMD	Bilateral carotid systems
			7	Female	Optic glioma; bilateral MMD	Bilateral ICA

*MMD, Moyamoya disease; MGDA, morning glory disc anomaly; BRVO, branch retinal vein occlusion; CRVO, central retinal vein occlusion; CRAO, central retinal artery occlusion; OS, oculus sinister; OD, oculus dexter; OU, oculus uterque; ICA, internal carotid artery; MCA, middle cerebral artery; ACA, anterior cerebral artery; PCA, posterior cerebral artery; NA, not available*.

Retinal vascular occlusion can be recurrent in MMD patients. The patient in case 12 was diagnosed with bilateral MMD and received encephalo-myo-synangiosis as a surgical intervention. Six years after the surgery, the patient complained of decreased vision in the right eye, and branch retinal vein occlusion was found in this eye. Recurrence occurred in his contralateral eye 12 years later (24). Case 13 indicated that the treatment of MMD might provide an increased blood supply to the ophthalmic artery and improve retinal perfusion as a possible solution to rescue vision (25). The visual acuity of this patient was significantly improved from 20/200 to 20/25 after an uncomplicated superficial temporal artery–MCA anastomosis. Case 16 reported a pregnant patient of mitral valve prolapse combined with right eye central retinal artery occlusion (CRAO) and bilateral MMD (27). It is considered challenging to identify a mechanism of CRAO in MMD patients, which can be caused by complicated factors and interactions. The hypercoagulable state during pregnancy, the pathological consequence of MMD, and the emboli from mitral valve prolapse may all be presumably responsible for this case.

The steno-occlusive changes of MMD usually occur at the supraclinoidal portion of the ICA and its two main branches. In some circumstances, the slow progression of MMD allows collateral circulation from the external carotid artery to develop and supply the central retinal artery (30). Aside from patients with cardiovascular emboli, CRAO is generally seen in the elderly population, especially those over 50 years old. However, the average age in the cases of MMD-associated CRAO mentioned above showed a younger tendency (26). When steno-occlusive and chronic ischemic changes involve the ophthalmic artery in MMD patients without the formation of collateral circulation, though rare, retinal artery occlusion may eventually be a complication. As for retinal vein occlusion in MMD, whether it results from the intravascular inflammatory change and subsequent compression of the vein, or it is coincidental is still in controversy (24).

### Ischemia- and Hemorrhage-Related Visual Disturbance

Diverse ocular manifestations are sometimes consequences of ischemia or hemorrhage in MMD and may differ from the regions involved. A total of 10 cases from eight articles referring to ischemia- or hemorrhage-related ocular manifestations are summarized and reviewed, five of which are from case series (Table 2) (31–38). Most patients had a significant ischemia lesion in the cerebral imaging, and only one patient was diagnosed as having a hemorrhage. One patient in Case 23 did not have any suggestive change of ischemia or hemorrhage in imaging. She was found with recurrent amaurosis fugax, and cerebral angiography only showed bilateral narrowing of the ICA, ACA, and MCA (35).

Decreased cerebral blood flow is a prevalent pathological change in MMD with a tendency to cause cerebral hypoperfusion. Severe hypoperfusion may lead to cognitive impairment and intellectual disability (4). Case 19 reported a patient with cerebral hypoperfusion-related epilepsy and nystagmus. She complained of headache and transient hemiparesis on the left side and was subsequently diagnosed with right-side MMD, grade III of Suzuki's angiographic staging. Hypoperfusion in her right basal ganglia, frontal, and parietal regions were also found. After a hyperventilation test, which is a trigger of transient ischemic attack (TIA) in MMD patients, she deteriorated with new-onset epileptic nystagmus. In this case, the authors indicated that the decreased cerebral blood flow in the right frontal region was responsible for the epileptic nystagmus.

Hemorrhage may be a result of aberrant angiogenesis, such as fragile microaneurysms or false aneurysms, especially those occurring in collateral vessels of MMD (39). Case 20 described a patient who used to have an intracranial hemorrhage. Even after receiving a superficial temporal artery to MCA bypass, he still suffered from diplopia and hypertropia after the operation. His hypertropia and diplopia were considered as consequences of trochlear nerve palsy, for the past hemorrhage had damaged the right trochlear nucleus and intra-axial trochlear nerve (32).

Although posterior circulation is rarely involved in MMD, it should still be regarded as an alternative cause of acute vision decrease. The possible mechanism of posterior circulation involvement in MMD is similar to that of anterior circulation besides an isolated pathological change, the unstable hemodynamic state of the posterior cerebral artery (PCA) (37). Patients with PCA involvement are usually related to more severe conditions and worse prognosis (4, 40). Prior studies have reported that ~29% of MMD patients were with PCA involvement, and 17% of them had an infarction in territories of PCA (4). Patients with PCA involvement usually present with specific symptoms related to impaired vision. Steno-occlusion changes in the posterior choroidal arteries of deep PCA can result in sectoranopia, while superficial segments infarction of PCA frequently leads to visual field defects (41). According to our review, 12 cases of PCA involvement were identified with ocular abnormalities, including optic disc pallor, congenital cataract, transient visual field defect, and occipital lobe infarction-related cortical blindness (33, 34, 36, 37). Most of these patients received revascularization surgery, such as superficial temporal artery to MCA bypass, encephalo-duro-galeo-synangiosis, etc. However, the therapeutic effect on visual manifestations was uncertain. Three patients in case 26 experienced vision improvement, yet cortical blindness persisted in the patient of case 21. The regions of ischemic stroke, time from occurrence to surgery, territories of the cerebral vessels with the steno-occlusive change, and the general condition of patients may all be implicit factors in determining the therapeutic effect.

### Other Ocular Findings

Other uncommon ocular findings in MMD patients are referred to in this section from 12 articles, including optic nerve involvement, congenital cataract, and retinochoroidal anomaly (Table 2) (34, 38, 42–51). In Case 31, a 13-year-old female patient was diagnosed with congenital glaucoma and MMD. The author pointed out an overlapped genetic mutation responsible for moyamoya angiopathy, as well as Fanconi anemia, congenital glaucoma, and other congenital abnormalities (46). Case 34 reported two pediatric MMD patients with bilateral iris hypoplasia and patent ductus arteriosus (49). Various congenital diseases are suggested that simultaneously involve cerebral vascular dysplasia, cardiovascular malformations, and ocular anomalies. Further study is needed to focus on possible target genes responsible for the conditions that related to MMD, congenital ophthalmic diseases, and other congenital diseases.

Retinal vasculature is a microcirculation that can be non-invasively observed by fundus photography. It could also be considered as a reflection of the systemic circulation. Case 27 reported a 30-year-old female with headache and blurred vision who was diagnosed with retinal arterial tortuosity and bilateral MMD. Proangiogenic factor levels increase as compensation and lead to neovascularization, which is hypothesized as an underlying explanation for the formation of retinal vascular tortuosity in this case (42). Case 28 described a patient of ocular ischemic syndrome combined with MMD and received a graft surgery. Significant damage to the blood–retinal barrier persists despite postoperative cerebral reperfusion. Though the ocular ischemia is capable of being relieved after the surgery of MMD with improved retinal circulation times, visual acuity outcomes are in limited optimism (43). Some patients with MMD are still at the risk of having retinovascular abnormalities, yet without obvious ocular symptoms. J.I. Chung reported a case of an MMD patient with ophthalmic rete mirabile, a plexiform vascular network of a reconstituted ophthalmic artery which is rarely seen in humans (52). The complex of the abnormal ophthalmic artery and its collaterals could be considered as embryonic remnant anastomosis and annexations and, thus, to some extent, explain the cause of prenatal cerebral vascular occlusion in MMD.

## Conclusion

MMD is associated with varying ophthalmic findings, particularly MGDA, according to previous studies. The risk of having MMD in patients of MGDA is higher than in the general population. Deficiency during neuroectodermal genesis and the subsequent mesodermal changes may be responsible for this association. Cerebral vascular examinations are recommended for MGDA patients to exclude any potential life-threatening intracranial vascular diseases. Other ophthalmic findings related to MMD include retinal vascular occlusion, visual field defects caused by ischemia or hemorrhage, optic disc abnormalities, etc. Retinal examinations would be beneficial for MMD patients to prevent severe loss of vision. Posterior circulation involvement in MMD is rare but should not be underestimated, for sometimes it is responsible for acute or transient vision loss. Both neurologists and ophthalmologists should recognize the correlation between ocular manifestations and cerebrovascular diseases such as MMD, in order to achieve a comprehensive diagnosis.

## Author Contributions

KY, JC, and YW designed this review. YW and KZ drafted the manuscript and searched and reviewed the database and all included articles. YY and FS reviewed the articles based on inclusion and exclusion criteria. YW, KZ, YY, FS, JY, JC, and KY provided comments and revised the manuscript. All authors have approved the final version of the manuscript.

## Conflict of Interest

The authors declare that the research was conducted in the absence of any commercial or financial relationships that could be construed as a potential conflict of interest.

## Abbreviations

MMD: moyamoya disease
ICA: internal carotid artery
MCA: middle cerebral artery
ACA: anterior cerebral artery
MGDA: morning glory disc anomaly
CRAO: central retinal artery occlusion
TIA: transient ischemic attack
PCA: posterior cerebral artery.
